# Rapid and Highly Sensitive Non-Competitive Immunoassay for Specific Detection of Nodularin

**DOI:** 10.3390/microorganisms5030058

**Published:** 2017-09-12

**Authors:** Sultana Akter, Markus Vehniäinen, Harri T. Kankaanpää, Urpo Lamminmäki

**Affiliations:** 1 Molecular Biotechnology and Diagnostics, Department of Biochemistry, University of Turku, FI-20520 Turku, Finland; marvehni@utu.fi (M.V.); urplammi@utu.fi (U.L.); 2 Marine Research Centre, Finnish Environment Institute, Hakuninmaantie 6, FI-00430 Helsinki, Finland; harri.kankaanpaa@ymparisto.fi

**Keywords:** nodularin, cyanotoxin, hepatotoxin, non-competitive immunoassay, quantification of nodularin, anti-immunocomplex binder, synthetic antibody phage library

## Abstract

Nodularin (NOD) is a cyclic penta-peptide hepatotoxin mainly produced by *Nodularia spumigena*, reported from the brackish water bodies of various parts of the world. It can accumulate in the food chain and, for safety reasons, levels of NOD not only in water bodies but also in food matrices are of interest. Here, we report on a non-competitive immunoassay for the specific detection of NOD. A phage display technique was utilized to interrogate a synthetic antibody phage library for binders recognizing NOD bound to an anti-ADDA (3-Amino-9-methoxy-2,6,8-trimethyl-10-phenyldeca-4(E),6(E)-dienoic acid) monoclonal antibody (Mab). One of the obtained immunocomplex binders, designated SA32C11, showed very high specificity towards nodularin-R (NOD-R) over to the tested 10 different microcystins (microcystin-LR, -dmLR, -RR, -dmRR, -YR, -LY, -LF, -LW, -LA, -WR). It was expressed in *Escherichia coli* as a single chain antibody fragment (scFv) fusion protein and used to establish a time-resolved fluorometry-based assay in combination with the anti-ADDA Mab. The detection limit (blank + 3SD) of the immunoassay, with a total assay time of 1 h 10 min, is 0.03 µg/L of NOD-R. This represents the most sensitive immunoassay method for the specific detection of NOD reported so far. The assay was tested for its performance to detect NOD using spiked (0.1 to 3 µg/L of NOD-R) water samples including brackish sea and coastal water and the recovery ranged from 79 to 127%. Furthermore, a panel of environmental samples, including water from different sources, fish and other marine tissue specimens, were analyzed for NOD using the assay. The assay has potential as a rapid screening tool for the analysis of a large number of water samples for the presence of NOD. It can also find applications in the analysis of the bioaccumulation of NOD in marine organisms and in the food chain.

## 1. Introduction

Cyanobacterial toxins pose a threat for humans, and toxin-contaminated water bodies have accidentally been utilized for recreational use, as a supply for drinking water and production of seafood. Domestic and wild animals have been affected and even killed because of cyanobacterial toxins (for a review, see [1]). Furthermore, the bioaccumulation of cyanotoxins like nodularin (NOD) in the aquatic food web to seafood such as flounder, cod, clam and mussel has been confirmed [2,3,4,5]. The first identified and scientifically reported cyanobacteria to cause animal poisonings was *Nodularia spumingena* from Australia in 1878 [6], although the plausible toxin, NOD, was identified and its structure resolved about 110 years later [7,8]. NOD is a non-ribosomally composed cyclic pentapeptide and shares a structural similarity, like a non-proteinogenic amino acid, with ADDA side chain, to microcystins (MCs)—the most frequently occurring and widespread cyanotoxin group. NOD, like MCs, block protein phosphatase 1 and 2A function [9,10,11] and has been characterized as hepatotoxic [12] and as possible tumor promoter [13,14]. NOD causes oxidative stress in mammals [15,16], plants [17,18], and fish [19,20,21]. Though about 10 analogues of NOD are reported in the literatures [22], NOD-R is the most abundant and often referred simply as NOD.

Only a few cyanobacteria species have been associated with NOD synthesis. The main producer is *N. spumigena*, which is found in brackish waters and low salinity coastal water bodies in different continents [23]. Frequently *N. spumigena* forms blooms in the brackish Baltic Sea [24,25] and in several brackish lakes [26]. Terrestrial *Nostoc* sp. cyanobacteria have been reported to produce NOD in symbiotic associations [27]. Another species with reported capacity to produce NOD is a benthic *Nodularia sphaerocarpa* [28,29], which was identified from a French thermal spring. Novel NOD-producing cyanobacterium has recently been characterized from a freshwater ambient spring in Queensland, Australia [30] with proposed new cyanobacteria genus and species name *Iningainema pulvinus* gen. nov., sp. nov.

Analytical methods to detect even minor amounts of NOD are very important to avoid exposure of contaminated water or food to humans and animals. Several approaches for simultaneous detection of hepatotoxins (MCs and NOD) have been described since the 1980s and include mouse bioassay, protein phosphatase inhibition assays, high-performance liquid chromatography (HPLC)-based methods coupled to optical detector or different mass spectrometry (MS) detectors, and immunoassays (ELISA). Mouse bioassay [31], protein phosphatase inhibition assays [32] and most immunoassays are not NOD specific and may respond to other toxins present in the sample. HPLC-based methods require special expertise and instrumentation, but still the liquid chromatography coupled to MS detectors have been the most promising tool to selectively analyze different hepatotoxin variants, and they can detect even the minor variants with respectable sensitivity [33]. Immunoassays can provide better sensitivity than LC systems and are extremely useful for the screening of a large number of potentially hazardous samples due to their simplicity and inexpensiveness. However, acquiring a NOD-specific antibody that is essential for the immunoassay has been a cumbersome process. Several attempts have led to antibodies recognizing NOD and MCs with varying affinities [34,35,36,37,38,39]. Two successful immunizations and monoclonal antibody selections have been reported with a NOD-specific antibody to develop competitive immunoassay of NOD. The announced analytical detection limits in these reports were 0.2 µg/L [35] and 0.16 µg/L [40].

We have recently established a broad specificity non-competitive immunocomplex assay for MCs and NOD [41]. This one-step immunocomplex assay was fast and highly sensitive with a detection limit below 0.1 µg/L for all ten tested MC variants and NOD within one hour assay time. The assay was based on the use of two antibodies with special binding characteristics. An anti-ADDA Mab was used to capture the toxins (MCs or NOD) in a generic manner, and the thereby-formed immunocomplex was recognized by the binder SA51D1, derived from a synthetic antibody library by phage display.

Encouraged by the success with the generic assay, we questioned whether a similar approach could be used to establish a non-competitive assay specific for NOD. Using NOD-R to form the immunocomplex with the anti-ADDA specific Mab and phage display selection from the synthetic antibody library, we show here the isolation of NOD-specific immunocomplex binders and the development of the first non-competitive NOD-specific immunoassay. The applicability of the assay for the specific detection of NOD is demonstrated from different types of solid-phase samples including fish tissue and several different natural water-phase samples.

## 2. Materials and Methods

### 2.1. Common Materials and Instruments

Common inorganic and organic chemical reagents were obtained from commercial source either from Sigma or Merck unless otherwise specified. Organic solvent methanol was of analytical or chromatographic grade and purchased from BDH Prolabo VWR chemicals (Radnor, PA, USA) and Fisher Scientific (Leicestershire, UK). The water used was purified by Millipore Milli-Q Plus water filtration purification system (Millipore Corporation, Billerica, MA, USA). Restriction enzymes were either from Fermentas (Vilnius, Lithuania) or from New England Biolabs (Ipswich, MA, USA). Oligonucleotides were custom-synthesized by Tag Copenhagen or biomers.net. Molecular biology techniques were performed according to the standard protocols [42] if not mentioned. DNA manipulation kits were from Qiagen (Hamburg, Germany) or from Gene Jet (Fermentas Life Sciences, Vilnius, Lithuania). Streptavidin-coupled magnetic beads (Dynabeads^®^ MyOne™ Streptavidin C1 and Dynabeads^®^ M-280 Streptavidin) and Dynal MPC magnet were purchased from Invitrogen, Thermo Fisher Scientific (Waltham, MA, USA). The fluorescence of Eu-chelate label was measured with Victor 1420 Multilabel Counter (Wallac/PerkinElmer Life Sciences, Waltham, MA, USA) using 340 nm excitation filter, 616 nm emission filter, 400 µs delay and 400 µs measurement time. Assay buffer was composed of 100 mM TSA (100 mM Tris, 0.9% NaCl, 0.05% NaN_3_ buffer) at pH 7.75 supplemented with 0.02% Tween 40, 0.1% Bovine-γ-globulin, 40 µM DTPA (diethylenetriaminepentaacetic acid), 1% Bovine serum albumin (BSA) and optional 20 µg mL^−1^ of Amaranth dye solution (CAS: 915-67-3, Sigma) to aid pipetting. Wash buffer for washing of microtiter well-plate contained 5 mM Tris-HCl pH 7.75, 0.9% NaCl, 0.1% Germall II, and 0.005% Tween 20. Europium fluorescence intensifier (EFI) solution, and streptavidin (low fluorescence background) or anti-mouse-IgG coated microtiter plates were from Kaivogen Oy (Turku, Finland). The monoclonal antibody AD4G2 (ADDA specific, anti-MCs) was purchased from Enzo Life Sciences, Inc. (Farmingdale, NY, USA) and was biotinylated [41] to be captured on the streptavidin surface. Bacterial anti alkaline phosphatase (AP) polyclonal antibody (anti-AP pAb) was purchased from, LifeSpan Biosciences Inc. (Seattle, WA, USA) and was labeled with europium [41] to use it as tracer reagent in the assay. Eu-labeled anti-phage Mab 9E7 was produced at the department of Biochemistry/Biotechnology, University of Turku. Histidine tag scFv purification was done with His Spin Trap™ kit (GE Healthcare, Little Chalfont, UK). DNA and protein concentration were measured by NanoDrop ND1000 spectrophotometer (Thermo Fisher Scientific, Waltham, MA, USA).

### 2.2. Toxin Standards

Specific amounts of the purified toxins: microcystin-LR (MC-LR), 3-desmethyl microcystin-LR (MC-dmLR), microcystin-RR (MC-RR), 3-desmethyl microcystin-RR (MC-dmRR), microcystin-LY (MC-LY), microcystin-LF (MC-LF), microcystin-LW (MC-LW), microcystin-YR (MC-YR), and nodularin-R (NOD-R) were obtained from Dr. Jussi Meriluoto’s Lab (Åbo Akademi University, Turku, Finland) as a lyophilized dried powder. Toxins had been purified as described earlier [43]. Microcystin-LA (MC-LA) and microcystin-WR (MC-WR) were purchased from Enzo Life Science (Farmingdale, NY, USA). All toxin standards were stored dry at −20 °C until required. Dry powder was dissolved in 50% methanol (100–250 µg/mL original stock) from which further working stocks and standards were prepared with reagent water. These dilutions were also stored at −20 °C or 4 °C.

### 2.3. Affinity Selection of Anti-Immunocomplex NOD-Specific Phage Antibodies

A synthetic scFv phage library termed scFvP [44,45] was used for the phage display-based selection of anti-immunocomplex NOD-specific binder. The selection protocol is the same as we described earlier [41] with the exception that in this study NOD-R was used instead of MC-LR. Phages were enriched with two panning rounds. Phage enrichment during the selections was followed by phage immunoassay by the method described earlier [41]. In short, biotinylated anti-ADDA Mab was immobilized on streptavidin-coated microtiter wells and immunocomplexes were formed by addition of free toxins (MC-LR, MC-RR or NOD-R). Then enriched phages (2e10 phage) from each panning rounds were added and bound phage were detected with Eu-chelate labeled anti-phage Mab.

### 2.4. Cloning, Expression, Screening and Purification

The pool of scFv genes isolated from the second selection round was transferred into vector pLK06H [45] for the expression of scFv as a fusion to alkaline phosphatase and histidine-tag (scFv-AP) in XL1 *E. coli* cells. To produce individual scFv clones, colonies of transformed cells were used to inoculate expression cultures in the wells of 96-well culture plates as described earlier [41].

After freezing and thawing of expression cultures (twice) and centrifugation, the culture supernatant was used for the screening immunoassays. Anti-ADDA Mab (20 ng/50 µL/well) captured on anti-mouse-IgG coated microtiter wells was incubated with NOD-R (concentration in reaction well: 10 µg/L) to form the immunocomplex followed by addition of the samples containing the culture supernatant. After 1 h incubation and washing, the AP activity was measured using pNPP substrate to detect the presence of the active anti-immunocomplex scFv-AP in the sample as described earlier [41]. In a similar manner, the positive clones were tested again for their specificity towards MC-LR, MC-RR and NOD-R. Clones, which showed activity towards NOD-R and not to the MC-LR and MC-RR, were expressed in a 5 mL culture volume and their specificities were tested towards nine different toxin variants (MC-LR, -dmLR, -RR, -dmRR, -YR, -LY, -LF, -LW, and NOD-R) in a similar manner. The selected NOD-specific clones were sequenced (by Macrogen Ltd., Amsterdam-Zuidoost, The Netherlands). Finally, the scFv-AP clone SA32C11 was expressed in 50 mL culture, purified by immobilized metal affinity chromatography column (His Spin Trap™ kit, GE Healthcare, UK) according to the manufacturer’s instructions and used in the assay development.

### 2.5. The Non-Competitive NOD-Specific Assay

The scFv-AP SA32C11 was used to develop a non-competitive assay. In a single step assay, 100 µL/well of reagent water (for blank measurement 10–24 replicates were used), NOD-R standard stocks (0.002–600 µg/L, prepared in reagent water), or samples were added in prewashed streptavidin wells (2–3 replicates for standard or sample). Then 100 µL/well of reagent mixture (biotinylated anti-ADDA Mab, 1 µg/mL; scFv-AP, 1 µg/mL; and N1-Eu-anti AP pAb, 0.5 µg/mL prepared in assay buffer) were added to the wells. The wells were incubated for 1 h (RT, slow shake) followed by four washes. Then EFI solution was added (200 µL/well), incubated for 10 min (RT, slow shake) and the fluorescence of Eu label was measured with Victor 1420 Multilabel Counter using time resolved fluorometry and Europium program. The detection limit (the smallest detectable toxin concentration in sample) was calculated from the standard curve based on the average response of replicates of blank + 3 times standard deviation of the blank. Concentrations of the unknown samples were calculated from the standard curve using Origin 2015 software (OriginLab Corporation, Wellesley Hills, MA, USA).

### 2.6. Effect of Incubation Time and Total Assay Volume

The effect of incubation time on the performance of the assay was observed. After addition of standard (100 µL) and reagent mixture (100 µL) the wells were incubated for 5 min to 4 h before washing step.

The 1 h incubation assay was also performed in 100 µL total reaction volume using (1) 50 µL of standard/sample and 50 µL of reagent mixture; and (2) 25 µL assay buffer + 25 µL sample/standard + 50 µL reagent mixture. 

### 2.7. Analysis of the Specificity Profile of SA32C11

NOD-R and ten different purified MC analogues (MC-LR, -dmLR, -RR, -dmRR, -YR, -LY, -LF, -LW, -LA, -WR) were analyzed to characterize the specificity profile of the purified scFv-AP SA32C11 by the immunocomplex assay in 100 µL total reaction volume as follows. In prewashed streptavidin strips, 25 µL of assay buffer were added followed by addition of 25 µL/well of different toxin standard solution of 0.02 to 600 µg/L. Then 50 µL/well of reagent mixture (1 µg/mL of biotinylated anti-ADDA Mab, 1 µg /mL of scFv-AP SA32C11 and 0.5 µg/mL of N1-Eu-anti bAP PAb) were added to the well (toxin concentration in reaction well becomes: 0.005 to 150 µg/L). The wells were incubated (1 h), washed four times, EFI solution was added (200 µL/well), and Eu fluorescence signal was measured after 10 min as in 2.5. 

### 2.8. Sample Analysis

The assay was applied to analyze a set of environmental water and bloom samples. The information regarding these samples is shown in Table 1.

#### 2.8.1. Recovery of NOD from Spiked Water Samples

Several different spiked water samples including reagent water, tap water, sea and coastal inlet water were analyzed with the non-competitive NOD-specific assay. The sea and coastal inlet water samples were collected in 2010 at the Åland islands, Finland and stored as such without any treatment at −20 °C. After thawing at room temperature, these water samples were used for spiking experiment without any filtration. Non-spiked environmental samples were tested with the generic assay [41] at the beginning. For each sample, spiking was done as follows: 40 µL of 600 µg/L NOD-R standard stock was added to 3.96 mL water sample to obtain 6 µg/L spiked solution, from which solutions of 3, 1, 0.3 and 0.1 µg/L were prepared by serial dilutions. All non-spiked and spiked (3, 1, 0.3, 0.1 µg/L) samples were analyzed by the assay using NOD-R as standard. The water samples were used in the immunoassay directly without any concentration or dilution steps.

#### 2.8.2. Raw Water Samples with Known Toxin Content

As a control, thirteen lake water samples and two seawater samples from Finland and Estonia (Table 1) were tested by the NOD-specific immunoassay using 100 µL total volume (50% sample and standard). For these samples, total toxin concentrations (cell extracted) as well as the toxin variants identified by LC-MS were already known [41,47,48]. All lake water samples were NOD-negative and most of them were positive for microcystins. The two seawater samples were NOD-positive. In addition, as a negative control, tap water from the laboratory was also tested by the immunoassays. The samples were stored at −20 °C and after thawing at room temperature (RT) they were used in the immunoassay as raw. Appropriate dilutions were prepared in reagent water.

#### 2.8.3. Sea and Coastal Inlet Water

Nine raw sea and coastal inlet water samples (Table 1) collected from Åland island Finland (sample locations: 2, 5, 6, 13, 15, 22, 30, 31, 32 published in supplementary, Savela et al., 2015) [46] were tested for MC/NOD for the first time in the present study. The samples were analyzed in 100 µL reaction volume by the generic assay [41] and the NOD-specific assay comprising 50 µL sample/standard + 50 µL reagent mixture. For both assay NOD-R was used as standard. 

#### 2.8.4. Bloom Samples from the Sample Archive 

A total of four Baltic Sea bloom samples from the sample archive of the Finnish Environment Institute, Marine Research Centre, were used (Table 1). These samples were collected from onboard the Finnish research vessel Aranda (owner Finnish Environment Institute) and had been stored at −20 °C since. They consisted of two surface bloom containing water samples (LL8 and LL19), and two freeze dried surface phytoplankton samples (Ajax 1 and LL3a). After thawing at room temperature and mixing thoroughly the LL8 and LL19 samples were used in the assay as raw. Several fold dilutions were prepared in reagent water. Ajax 1 and LL3a samples were processed as described in Section 2.8.6.

#### 2.8.5. Tissue Samples

Purchased fish fillet tissue and Baltic Sea fauna from the sample archive of the Finnish Environment Institute, Marine Research (Supplementary Materials Table S1) were analyzed by the NOD-specific assay as well as by the generic assay [41]. Tissue sample processing, analysis and results are described in the supplementary section.

#### 2.8.6. Extraction of Hepatotoxins

Toxin from the freeze-dried phytoplankton (Ajax1 and LL3a) samples and tissue samples (raw or freeze dried) were extracted in 70% (Table 1) and 100% methanol (Supplementary Materials Table S1) respectively. The methanolic extracts were stored at +4 °C, overnight (~16 h) in glass tubes and on the following day, they were sonicated in bath sonicator (Finnsonic m03; Finnsonic Oy, Lahti, Finland) for 1 h (temp 33 °C) for toxin extraction. Then the bottles were centrifuged at 4000 rpm for 20 min at 4 °C. Total extraction time was ~21 h before separation of solid phase from the supernatant. 1 mL methanolic extract aliquots from each sample were collected to 1.5 mL glass vial. From one sample set, the liquid (1 mL aliquot/vial) was evaporated to dryness with nitrogen gas at room temperature. Then 200 µL of reagent water was added to each vial and mixed thoroughly by vortexing and brief bath sonication (3 min). These samples were used as such and after appropriate dilution by generic immunoassay and the NOD-specific immunoassay. Due to low sample volume, immunoassay was performed in 100 µL total reaction volume comprising 25 µL sample/standard + 25 µL buffer + 50 µL reagent mixture (1 µg/mL of bio-anti-ADDA Mab, 1 µg/mL of scFvAP SA51D1 (generic assay) or SA32C11 (NOD-specific assay) and 0.5 µg/mL of Eu-anti-bAP).

## 3. Results

### 3.1. Immunocomplex Panning

An in-house synthetic antibody phage library scFvP [45] was exploited to isolate the antibodies recognizing the immunocomplex (IC) of NOD-R bound to anti-ADDA Mab. Biotinylated anti-ADDA Mab was immobilized on the surface of streptavidin-coated magnetic beads and NOD-R was added in excess to form a Mab:NOD immunocomplex. The beads were used for the selection of the binders from the synthetic scFv library displayed on the phage. Two rounds of selection (panning) by phage display were performed and subtractive pannings were done to remove binders with specificity towards streptavidin or naked anti-ADDA Mab. The phage rescued at different panning rounds were tested for their immunoreactivity towards immobilized anti-ADDA Mab saturated either with MC-LR, MC-RR or NOD. After the two panning rounds, the phage library showed 54-fold higher signal when tested against immunocomplex than against naked anti-ADDA Mab, indicating an efficient enrichment of the binders. Interestingly, the phage library showed immunoreactivity towards all the three immunocomplexes, as we have seen earlier in the selections against immunocomplex comprising MC-LR [41], although the binding to MC-LR and MC-RR immunocomplexes was somewhat less pronounced compared to NOD immunocomplex. Some unwanted binders towards free anti-ADDA Mab were also enriched, but the enrichment was considerably weaker than against the Mab: NOD-R immunocomplex.

### 3.2. Screening Summary

The pool of scFv genes from the second panning round was cloned from the phage display vector to an expression vector for production of soluble scFv alkaline phosphatase fusion proteins (scFv-AP). After scFv-AP expression, a total of 360 individual clones were screened for binding towards the immunocomplex of NOD-R and anti-ADDA Mab immobilized on anti-mouse antibody coated wells. The binding of scFv-AP was detected by alkaline phosphatase activity after 60 min signal generation. 46% of the scFv-AP clones were positive (signal/blank, S/B > 3). The maximum S/B was 8.6 and a total of 37 clones had a S/B value of 5 or more.

Based on the best S/B ratio, the top 24 clones were further tested for their binding capacity towards immunocomplexes consisting of MC-LR, MC-RR and NOD-R. This led to the shortlisting of 10 clones, and all the ten clones were found to be specific towards NOD-R: anti-ADDA-Mab immunocomplex after testing their cross reactivity in immunocomplex with nine different cyanotoxins (MC-LR, -dmLR, -RR, -dmRR, -YR, -LY, -LF, -LW, and NOD-R) at toxin concentration 10 µg/L in reaction well. DNA sequencing finally revealed nine unique clones which were further tested for their cross reactivity towards the above mentioned nine toxin variants at concentration range of 0.3 to 30 µg/L in the non-competitive immunoassay. Finally clone SA32C11 was selected and purified as scFv-AP with affinity chromatography. 

### 3.3. Specificity Profile of SA32C11 scFv-AP

**Figure 1:**
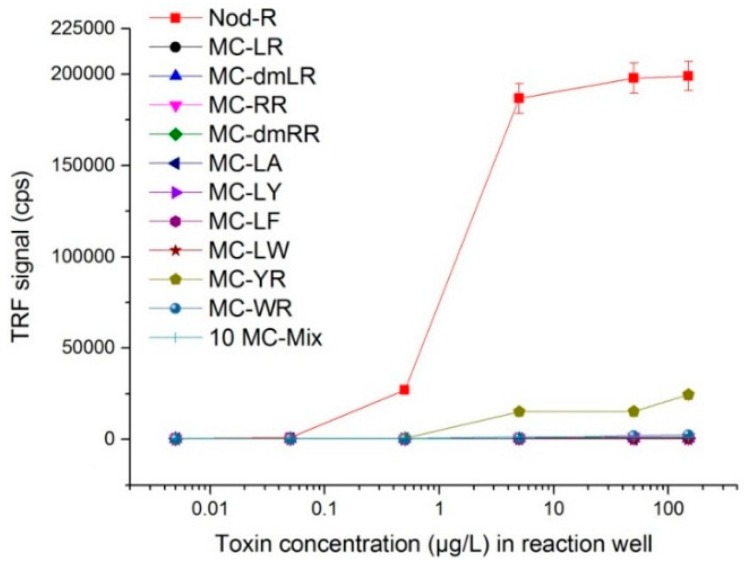
Cross-reaction profile of the anti-immunocomplex binder scFv-AP SA32C11 towards different cyanotoxins. The error bars represent the standard errors of means in the time-resolved fluorescence signals from duplicate measurements. The sample “10 MC-Mix” consists of an equimolar mixture of all the individually tested MCs (i.e., NOD not included). TRF: Time-resolved fluorescence. Cps: counts per second.

The purified scFv-AP clone SA32C11 was characterized more closely to reveal the specificity profile in the non-competitive assay format (Figure 1). Immunocomplexes were captured on the streptavidin surfaces and detected by Eu-labeled anti-alkaline phosphatase antibody. The scFv-AP showed a distinct specificity towards NOD-R and clearly detectable signal already at concentration range 0.1 to 1 µg/L. The scFv-AP showed some affinity (% signal than that of NOD-R at the maximum tested toxin concentration of 150 µg/L) towards the immunocomplex of MC-YR (12%), MC-WR (1.3%), MC-RR (0.7%), MC-dmRR (0.6%) and MC-LR (0.4%). The binding towards the rest of the MC analogues (MC-dmLR, MC-LA, MC-LY, MC-LF and MC-LW) or the free anti-ADDA Mab was below detection limit. 

### 3.4. Optimization of the Assay Time 

**Figure 2:**
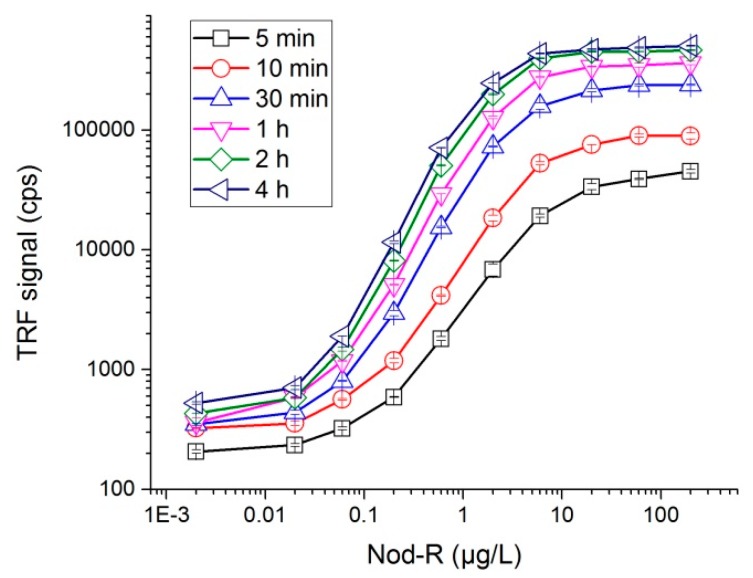
The dose-response curves for the single-step non-competitive assay with different reaction times. Each data point represents the average of duplicate measurements and the standard errors of the means are shown with error bars. TRF: Time-resolved fluorescence. Cps: counts per second.

In order to study the influence of the incubation time on the assay performance, bio-affinity reactions of different length were performed in a 200 µL reaction volume using 100 µL of NOD-R standard solution in concentrations from 0.002 to 200 µg/L. Figure 2 shows the dose-response curves for the different time points. The detection limit values calculated from the data (average blank + 3SD, *n* = 15–16) ranged from 0.1 µg/L to 0.03 µg/L within 5 min to 1 h reaction time. Signal level continued to slightly increase when the incubation was extended up to 2–4 h. However, the extended incubation did not improve the detection limit as the background signal also increased proportionally. Hereby, 30 min to 1 h was found to be the optimum incubation time to achieve best sensitivity. Alternatively, a reasonably good sensitivity could be obtained in a total assay time of 15 min (consisting 5 min bio-affinity reaction + 10 min incubation for fluorescent signal development).

### 3.5. Assay Performance at Different Volumes

**Figure 3:**
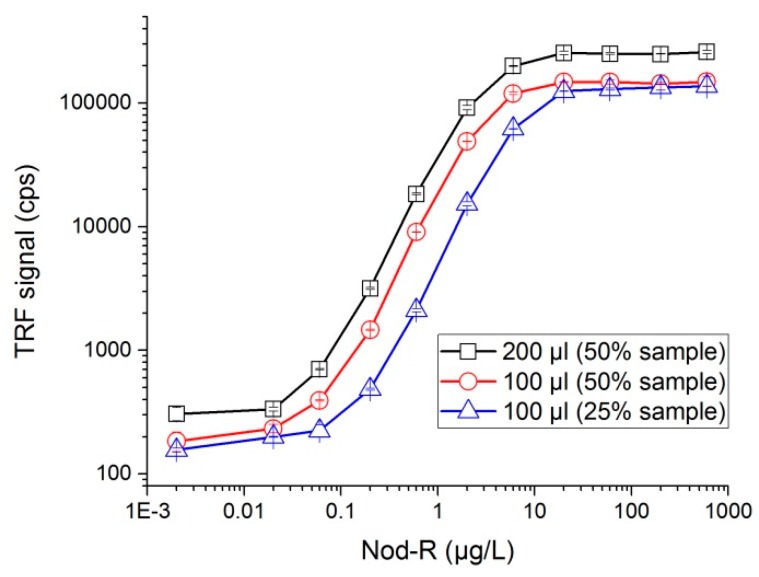
The dose-response curves for the single-step non-competitive assay with different reaction volume. Each data point represents the average of duplicate measurements and the standard errors of the means are shown with error bars. TRF: Time-resolved fluorescence. Cps: counts per second.

The assay was performed in total (1) 200 µL reaction volume comprising 50% of sample and also in 100 µL reaction volume comprising (2) 50% or (3) 25% sample volume (Figure 3). Analytical detection limit for NOD-R in these three conditions were 0.03, 0.06 and 0.1 µg/L, respectively.

### 3.6. Sample Analysis

#### 3.6.1. Recovery of NOD from Spiked Water Samples

A total of five water samples (including reagent, tap, sea and coastal inlet water) were spiked with NOD-R (0.1 to 3 µg/L) to evaluate the assay performance in detecting the NOD content in different water matrices. The recovery percentage ranged from 79 to 127% (Table 2). The unspiked samples were found to be free of MCs and NOD by generic assay [41] and the established NOD-specific assay. The coefficient of variation percentage (cv %) of the measurements was below 8.45. At the lowest spiked concentration (0.1 µg/L), the recovery ranged from 84% to 115% and the cv % of the measurements ranged from 1.68 to 4.62.

#### 3.6.2. Water Samples with Known Toxin Content

Fifteen raw surface water samples (thirteen lakewaters and two seawaters) collected in 2009 were analyzed by the NOD-specific assay (Table 3). For these samples extracted total toxin concentrations (MC-LR equivalent) had been previously analyzed by LC-MS and published earlier [47]. Also the toxin analogues in these samples were known [41,48]. The lakewaters contained no NOD but most of them contained MCs. On the other hand, both the seawater samples were MC negative and NOD-positive. According to the NOD-specific assay, toxin concentrations in the lakewater samples were below 0.09 µg/L, except for Savojärvi lakewater showing the toxin concentration value of 0.46 µg/L. The measured NOD-R levels in the seawater samples were 0.19 (Stroomi rand (Sea), Estonia) and 1.26 µg/L (Nåtö vägbank, (Sea), Åland, Finland). Tap water was also tested and contained no detectable NOD.

#### 3.6.3. Samples with Unknown Toxin Content

##### Sea and Coastal Water Samples

**Figure 4:**
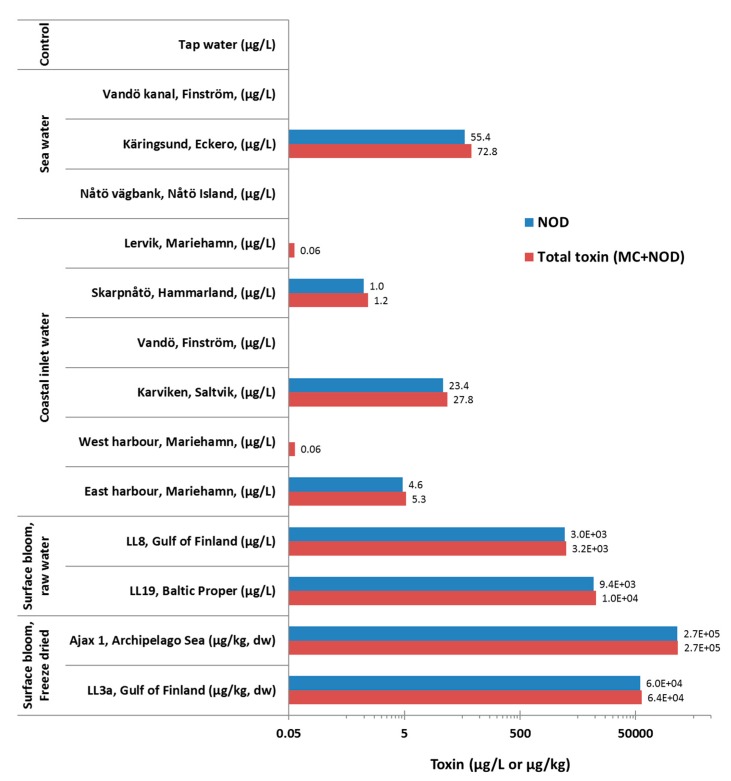
Concentration of NOD and total toxin (MCs and NOD) in environmental water and surface bloom samples. The total toxin concentration was measured with the generic assay [41] using NOD-R as standard.

Three seawater samples and six coastal inlet water samples collected in 2010 from Åland island Finland [46] were tested for the presence of NOD (Figure 4). Two seawater (Nåtö vägbank, Nåtö Island and Vandö kanal, Finström) and one coastal inlet water (Vandö, Finström) contained no detectable NOD. These samples were also negative according to the generic immunoassay [41]. Among the tested samples, Käringsund, seawater contained the highest NOD concentration of 55 µg/L. The total toxin concentration (NOD as standard) reveled by the generic assay showed similar or slightly higher concentration of total hepatotoxins in these tested samples. 

##### Surface Bloom Samples

Two untreated surface bloom water and two freeze-dried, aqueous-methanol-extracted phytoplankton materials were tested. The initial signal generated by the samples was very high and hence several fold dilutions were tested in the subsequent assay. NOD concentration of 2.9 × 10^3^ and 9.4 × 10^3^ µg/L were determined for the bloom water samples originating from the Gulf of Finland and the Baltic, Proper, respectively (Figure 4). For these two samples, total MC and NOD concentrations of 3.2 × 10^3^ and 10.4 × 10^3^ µg/L were measured, respectively. The freeze-dried, aqueous-methanol-extracted phytoplankton materials contained 6.0 × 10^4^ to 2.7 × 10^5^ µg NOD /kg, dw and 6.4 × 10^4^ to 2.7 × 10^5^ µg total hepatotoxin /kg, dw. 

##### Tissue Sample

Various tissue samples, including raw fish, mussel and clam tissue (Supplementary Materials Table S1) were analyzed by the NOD-specific assay to determine NOD content. The results of the tissue samples are shown in Supplementary Materials Figure S1.

## 4. Discussion

We report here a non-competitive immunoassay for the specific detection of NOD. In order to develop the assay, we used a synthetic antibody phage library-based in-vitro selection strategy to isolate binders recognizing the immunocomplex of the NOD and an ADDA-specific monoclonal antibody. The strategy turned out to be very efficient and a number of potential binder candidates were rapidly identified. Several of these, including the clone SA032-C11 used for the assay development, showed very good capability to distinguish NOD from several structurally similar MC variants (Figure 1). Recently, we have successfully utilized a similar strategy to develop non-competitive immunoassays for generic recognition of the MCs and NOD bound to same anti-ADDA Mab [41]. Surprisingly, despite the small size of the NOD, its complex with the anti-ADDA antibody evidently provides different types of epitopes and a highly diverse repertoire of synthetic antibodies can be employed to produce binders against these epitopes for the detection of NOD either in a very specific or more promiscuous manner. These results also implicate that it might be possible to generate specific binders against the immunocomplex of various other cyanotoxin congeners containing the same ADDA group, and develop novel immunoassay-based tools for toxin identification. 

Immunoassays for the detection of small molecules such as NOD are generally based on competitive formats since, due to steric hindrance, small analytes’ core structures usually do not allow simultaneous binding of two different antibodies essential for the non-competitive sandwich-type assay. Our immunocomplex concept-based non-competitive assay shows several advantages over the competitive assays. The presence of binder reagents in excess confers rapid assay kinetics, and there is more flexibility in terms of reaction time and volume. In addition there is no need to produce a labeled NOD antigen conjugate and the immunocomplex assay is also not affected by the hook-effect. Our assay shows excellent sensitivity reaching a detection limit of 0.03 µg/L with maximal 200 µL reaction volume. Compared to the previously described NOD-specific competitive immunoassays [35,40], the non-competitive assay is about 5- to 6-fold more sensitive. If desired, the assay can also even be performed in ca. 15 min reaction time or in reduced reaction volume of 100 µL with the reasonably good detection limits of 0.1 µg/L and 0.06 µg/L, respectively.

For assay validation, water samples with known toxin concentrations were analyzed. The first test, performed with spiked water samples, showed acceptable recoveries and excellent CV % values. Furthermore, there was no indication of significant interferences with different water matrices. The assay was then challenged by a set of sea and lakewater samples, most of which contained various MCs as revealed by the previous LC-MS analysis [41,47,48]. Among a total of 15 samples, the assay was able to correctly detect the two NOD-containing samples in a good accordance with the concentrations measured by LC-MS (Table 3). Among the rest 13 samples containing MCs in different combinations, but no NOD, eight were tested negative, four provided very slight positive values (0.08–0.09 µg/L) and one was clearly positive (0.46 µg/L). The sample giving clear false positive value had the highest total toxin concentration (40.9 µg/L) and contained four MCs variants, including MC-didmLR (didemethyl-microcystin-LR) and MC-didmRR (didemethyl-microcystin-RR) not assessed in the specificity analysis. Overall, the positive values from NOD-negative samples are apparently due to the minor cross-reactivity of several MC congeners in the assay. All but one of the false positive results could be avoided by setting the practical detection limit of the assay to the level of 0.1 µg/L, corresponding ca. the mean of blank + 9 SD. Altogether, based on these validation tests, the assay is suitable for the sensitive quantification of the NOD in the water samples which are unlikely to contain high concentrations of MCs. Thus, the assay could be very useful e.g., in the Baltic Sea region where NOD is a dominant cyanobacterial toxin. The assay results should be verified for example by LC-MS especially if elevated concentrations of MCs are expected to exist in the sampling site.

The assay was also applied to the analysis of the several different types of previously unanalyzed but potentially NOD-containing samples. These included samples from sea/coastal inlet water, fish and clam as well as phytoplankton. The total toxin concentration in these samples was also analyzed with the generic non-competitive assay [41].

Among a total of nine marine and coastal inlet water samples collected at the Åland Islands in 2010 [46], four clearly NOD-positive samples were found. As expected, very similar toxin concentrations were observed with both the NOD-specific and generic immunoassay, which is in accordance with the previous observations showing that NOD-R is the main toxin in this area and the Baltic Sea as a whole [50]. These samples have previously been analyzed for the cyanobacterial genera by Savela et al. [46], and all the samples tested positive for NOD by us contained *Nodularia* according to their study. Two of the samples (Karviken and Kärinsund) containing the highest concentrations of NOD were in mesotrophic state at the time of sampling. Moreover, *Nodularia spumigena* was the main cyanobacterial genera in the Kärinsund sample (personal communication with Henna Savela). 

To demonstrate the capacity of the assay to quantitate NOD in samples potentially containing high toxin concentration, surface bloom-containing seawater and phytoplankton samples were analyzed. In both cases the undiluted sample initially yielded signal level at the higher end of the standard curve and eventually multiple dilutions down to 1:10,000 were used to obtain the quantitative result. Eventually, mg/L levels of toxin were measured in the raw Baltic Sea surface bloom samples. When evaluating the detected NOD levels in water, it should be taken into account that freezing and thawing of the samples may also have contributed to the release of cell-bound toxins to the liquid phase. In any case, a NOD concentration as high as 25 mg/L has been reported, for example, from the Gulf of Gdansk in southern Baltic Sea [51]. Following the typical pattern of abundance of *Nodularia spumigena* blooms, the phytoplankton bloom samples from the central Gulf of Finland (LL3a) and western Gulf of Finland (Ajax 1) were found to contain 6.0 × 10^4^ and 2.7 × 10^5^ µg NOD/kg dw, respectively. These concentrations are in the range of those found e.g., in phytoplankton collected in 2000–2002 and 2006–2007 (1 × 10^3^–6 × 10^6^ µg total hepatotoxin/kg dw), [49] suggesting that substantial quantities of NOD-R is retained in samples stored for long time.

In order to evaluate the capacity of the assay for the analysis of bioaccumulation of NOD, tissues samples from clam and fish were tested. Apart from the purchased fish fillets, the samples originated from the areas that are yearly affected by *Nodularia spumigena* bloom and where hepatotoxins NOD-R and also MC-LR have been found in tissue samples [4,5]. Considering the results of both the NOD-specific and generic immunoassay, NOD was clearly the dominant toxin in most of the samples. NOD was also found in all the samples disregarding the sample pretreatments (freeze-dried or raw). Only the purchased fish fillet samples tested negative, which was not a surprise and, therefore, served as a negative control. 

Overall, the NOD concentrations in the clam and flounder samples were in accordance with earlier observation. For example, NOD concentrations of 10–110 µg/kg dw in *Macoma balthica* have been reported earlier [49], and here we found 4.0–26.5 µg/kg ww. Interestingly, in the case of raw *Macoma*, relatively similar toxin levels were detected in both the solid tissue and the liquid fraction obtained after centrifugation treatment of the organism. Even if not allowing completely quantitative analysis, direct detection from the liquid phase could be a very attractive approach for screening purposes, since laborious sample processing (such as organic-solvent-based toxin-extraction) can be avoided. However, more studies are needed to explore the correlation between the liquid and solid phase toxin levels. 

## 5. Conclusions

A unique synthetic antibody-based immunocomplex binder allowed development of a noncompetitive immunoassay for the specific detection of NOD. Due to the exceptionally good sensitivity and selectivity and other benefits conferred by the noncompetitive assay format, the assay is expected to be a useful detection tool for the quantitative analysis of NOD. The assay can find applications as a simple research tool for the detection and quantification of NOD in water or other environmental samples. It might also facilitate the analysis of bioaccumulation of NOD into marine organisms and potential transfer of the toxin further down the food chain. Usage of the NOD-specific assay in combination with our previously described generic assay for MC and NOD would allow the sensitive measurement of the total toxin and NOD-R concentrations.

## Figures and Tables

**Table 1:** Analyzed environmental water and bloom samples.

	Sample	Collection Place	Year	Previous Analysis/Comments	Pretreatment/Sample Processing
1	Spiked sea waters (*n* = 2)	Åland Islands, Finland	2010	Samples were previously analyzed for cyanobacterial saxitoxin biosynthesis (sxt) genes and paralytic shellfish toxins [46]. No previous analysis of MC/NOD for these samples.	Two seawaters with no detectable toxin were spiked with known amount of NOD before analysis.
2	Lake waters (*n* = 13)(MC positive, NOD negative)	Finland and Estonia	2009	Toxin content and toxin analogues were detected earlier by LC-MS analysis [41,47,48]. No detectable nodularin, most lakewater contain MCs.	No pretreatment, stored at −20 °C, used in the assay as raw.
3	Sea waters (*n* = 2) (NOD positive)	Finland and Estonia	2009	Toxin content and toxin analogues were detected earlier by LC-MS analysis [41,47,48]. Both samples contained NOD.	As above
4	Sea (*n* = 3) and coastal inlet waters (*n* = 6)	Åland Islands, Finland	2010	Samples were previously analyzed for cyanobacterial saxitoxin biosynthesis (sxt) genes and paralytic shellfish toxins [46]. No previous analysis of MC/NOD for these samples.	As above
5	Surface bloom containing seawater (*n* = 2)	Monitoring station LL19 (Baltic Proper)	2003	None Cyanobacteria blooms occur in the area during late summer every year	Concentration on a 50 µm plankton net, stored at −20 °C, analyzed after thawing at RT.
		Monitoring station LL8 (Gulf of Finland)	2007	None Sporadic cyanobacteria blooms occur in the area.	No pretreatment. Stored at −20 °C, analyzed after thawing at RT.
6	Surface phytoplankton bloom sample (*n* = 2)	Monitoring station Ajax 1 (Archipelago Sea) [49]	2002	Sporadic cyanobacteria blooms occur in the area.	Freeze-dried and was stored at −20 °C. 25 mg sample was mixed with 5 mL of 70% MeOH.
		Monitoring station LL3a (Gulf of Finland)	2009	As above	Sample was collected using a pump and filtration. Freeze-dried and was stored at −20 °C. 11 mg sample was mixed with 2.2 mL of 70% MeOH.

Note: Samples 5 and 6 were from the sample archive of the Finnish Environment Institute, Marine Research Centre. RT: Room Temperature.

**Table 2:** The performance of non-competitive assay with NOD-R spiked water samples.

	Origin of Water Sample and Date of Collection	NOD-R Added to the Sample (µg/L)	NOD-R Determined by Non-Competitive Assay (µg/L)	CV (%) of the Measurement	Recovery (%)
1	Reagent water 27.5.2017	0	0	-	-
		0.1	0.08	1.68	84
		0.3	0.27	2.83	91
		1	0.92	2.40	92
		3	2.38	4.51	**79**
2	Drinking tap water 27.5.2017	0	0	-	-
		0.1	0.09	4.62	90
		0.3	0.29	1.70	98
		1	0.95	1.49	95
		3	2.86	8.45	95
3	Nåtö vägbank, Nåtö Island (sea) 2.8.2010	0	0	-	-
		0.1	0.11	2.22	105
		0.3	0.32	1.83	107
		1	1.06	1.92	106
		3	3.26	5.66	109
4	Vandö, Finström, Coastal inlet 3.8.2010	0	0	-	-
		0.1	0.10	1.81	98
		0.3	0.30	4.37	102
		1	1.06	3.60	106
		3	3.25	3.52	108
5	Vandö kanal, Finström, Sea 3.8.2010	0	0	-	-
		0.1	0.11	2.16	115
		0.3	0.33	1.32	109
		1	1.18	1.84	118
		3	3.81	1.36	**127**

Unspiked samples were also negative according to the generic assay [41]. CV: The coefficient of variation.

**Table 3:** Toxin concentration measured by the non-competitive NOD-specific assay (NOD-R equivalent) from the fifteen water samples. The total toxin levels (MC-LR equivalent) and main toxin variants revealed by the previous LC-MS analysis [41,47,48] are also shown.

	Place	Date	NOD-Specific Assay	LC-MS Analysis Adapted from [41,47,48]	
			Toxin Concentration (µg/L)	Toxin Concentration (µg/L)	Toxin Analogues
1	Nåtö vägbank, (Sea), Åland, Finland.	29.7.2009	1.26	1.50	**NOD**
2	Stroomi rand (Sea), Estonia.	18.8.2009	0.19	0.25	MC-dmRR, **NOD**
3	Brantsböle, Åland, Finland.	27.7.2009	0.08	21.40	MC-dmLR, **MC-LR**, MC-LY, MC-LW, MC-LF
4	Hauninen reservoir, Raisio, Finland.	17.8.2009	<dl	0.39	**MC-dmRR**, MC-RR, MC-dmLR, 1031,5
5	Lemböte byträsk, Lemböte, Åland Islands, Finland.	29.7.2009	<dl	0.32	**MC-YR**, MC-dmLR
6	Lake Peipus, Rannapungerja beach, Estonia.	25.8.2009	0.08	0.60	MC-dmRR, **MC-RR**, MC-YR, MC-dmLR, MC-LR
7	Lake Peipus, Mustvee beach, Estonia.	14.8.2009	<dl	0.20	MC-dmRR, **MC-RR**, MC-dmLR, MC-LR
8	Littoistenjärvi, Kaarina, Finland.	3.9.2013	<dl	0.50	MC-dmRR, **MC-RR**, MC-YR, MC-dmLR, MC-LR
9	Littoistenjärvi, Kaarina, Finland.	11.9.2009	0.09	3.70	MC-dmRR, **MC-RR**, MC-YR, MC-dmLR, MC-LR
10	Hauninen reservoir, Raisio, Finland.	14.7.2009	0.09	0.27	MC-dmRR
11	Hauninen reservoir, Raisio, Finland.	15.9.2009	<dl	0.86	**MC-dmRR**, MC-RR, MC-dmLR, 1031,5
12	Hauninen reservoir, Raisio, Finland.	29.9.2009	<dl	1.90	**MC-dmRR**, MC-dmLR, 1031,5
13	Hauninen reservoir, Raisio, Finland.	29.10.2009	<dl	0.68	**MC-dmRR**, MC-dmLR, 1031,5
14	Savojärvi, Pöytyä, Finland.	7.8.2009	0.46	40.90	MC-didmRR, **MC-dmRR**, MC-didmLR, MC-dmLR
15	Littoistenjärvi, Kaarina, Finland.	4.8.2009	<dl	nd	nd

MC (microcystin), NOD (nodularin), <dl (below detection limit), nd (not detected), major toxin analogues are shown as bold.

## Supplementary Materials

The following materials are available online at www.mdpi.com/2076-2607/5/3/58/s1, Table S1: Analyzed fish and other tissue samples, Figure S1: Analysis of fish and tissue samples with the NOD-specific assay.
