# Supplementary material for: Rapid and Highly Sensitive Non-Competitive Immunoassay for Specific Detection of Nodularin
Source: Microorganisms. 2017 Sep 12;5(3):58. doi: 10.3390/microorganisms5030058 (PMC5620649; doi:10.3390/microorganisms5030058)
Supplement: Supplementary file 1 [file microorganisms-05-00058-s001.pdf]

Supplementary Material

Rapid and Highly Sensitive Non-Competitive Immunoassay for Specific Detection of Nodularin

Sultana Akter, Markus Vehniäinen, Harri T. Kankaanpää and Urpo Lamminmäki

Table S1. Analyzed fish and other tissue samples.

	Sample	Collection Place	Year	Previous Analysis	Pre-Treatment/Sample Processing
1	Fish fillet	Purchased from local supermarket. Baltic origin. (1) Finnish Salmon (medallion), (2) Flounder (*Platichthys flesus*), (3) Northern Pike (*Esox lucius*), (4) Baltic Herring, (*Clupea harengus membras*), (5) Finnish Zander (*Sander lucioperca*).	2017	No previous analysis.	No freeze drying. 2.5 g (ww) raw fish sample was mixed with 2.5 mL of PBS buffer and homogenized (IKA T25 digital Ultra-Turrax, IKA®-Werke GmbH & Co. KG, Staufen im Breisgau, Germany). 1 g of homogenized paste (~0.5 g fish + ~0.5mL PBS) was mixed with 5 mL 100% MeOH (~0.5 g fish + ~5.5 mL liquid).
2	*Macoma balthica* clams	Archipelago Sea, Gulf of Riga, Gulf of Finland.	2004, 2005, 2006.	The NOD-R concentrations detected in *Macoma balthica* were close to the level (10–110 µg/kg dw) found earlier in other samples of the same species [1].	No freeze drying. Collection of soft tissue from 10 clams. Soft tissue and raw liquid phase was separated by centrifugation (4000 rpm, 15 min, +4 °C). 5.7 to 6.5 g tissue (ww) was mixed with 1 mL of PBS and homogenized. 1 g homogenized paste was mixed with 5 mL of 100% MeOH (~0.85 g tissue + ~5.15 mL liquid). The separated liquid phase was diluted (1:10) to assay buffer and used in the immunoassay as such.
3	*Mytilus edulis*	Western Gulf of Finland. Collected from the wreck of Eira by diver from depth of 25–28 m.	1999	No analysis since the work made for the publication.	Freeze-dried and homogenized; was stored at −20 °C. 25 mg material was mixed with 5 mL of 100% MeOH.
4	Flounder liver	Composite sample of flounder livers from the Gulf of Finland collected between 2000 and 2005.	2005	None	Freeze-dried and homogenized. Was stored at −20 °C or 4 °C. 54 mg liver was mixed with 5.4 mL of 100% MeOH.

Note: Sample 2 to 4 were from the sample archive of the Finnish Environment Institute, Marine Researech Centre.

Analysis of tissue samples

Method

Five different raw fish fillet samples (Table S1) were purchased from the local supermarket (K-Citymarket Kupitta, 21.3.2017) and stored at +4 °C. The samples were processed within three days.

In addition, raw and freeze dried tissue samples (Table S1) from the sample archive of the Finnish Environment Institute, Marine Researech Centre were analyzed by the NOD specific assay. Among the samples were *Macoma balthica* clams from the Gulf of Finland (2006), the Archipelago Sea (2004) and the Gulf of Riga (Baltic Sea, 2005). These clams were stored as such at −20 °C and processed after thawing at room temperature. The samples also included homogenate of *Mytilus edulis* collected from the surface of a shipwreck (SS Eira, western Gulf of Finland), flounder liver (freeze dried and homogenized composite sample of flounder livers from the Gulf of Finland collected between 2000 and 2005). The samples originated from areas that are yearly affected by *Nodularia spumigena* blooms and where hepatotoxins (NOD-R and also MC-LR) are found in tissue samples [2]. The majority of the Baltic Sea samples bank specimen had not been analyzed for hepatotoxins earlier and none of the samples were analyzed in other laboratory before the present study.

For sample pretreatment and processing see Tables S1

To extract the hepatotoxin specific amount (Supplementary Materials Tables S1) of freeze dried samples or homogenized tissue paste samples were mixed with 100% methanol and stored at +4 °C overnight (~16 h) in glass tubes. On the following day, the methanolic extracts were handled in a similar way by method described in Section 2.8.5.

Results

The purchased fish samples contained no detectable toxin according to the nodularin specific as well as the generic assay (Supplementary Materials Figure S1). The NOD concentration in the *Macoma* samples ranged from 4.0 to 26.5 µg/kg, ww. Freeze-dried flounder liver and *Mutilus edulis* contained 10.7 and 45 µg/kg, dw of NOD by the NOD specific assay.

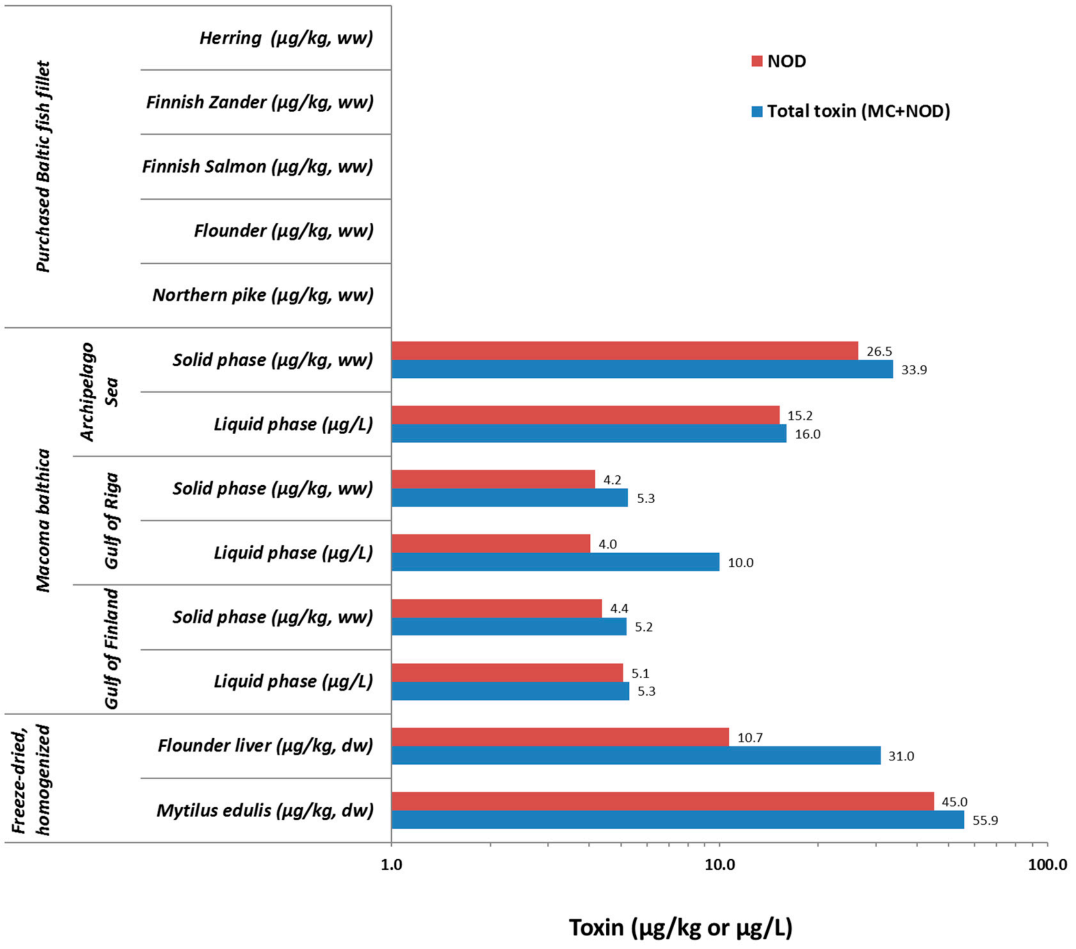

Figure S1. Analysis of fish and tissue samples with the NOD specific assay. The samples were also tested with the previously reported generic assay [3] using NOD-R as standard. Toxin concentration of the fish tissue samples purchased from supermarket was below the detection limit.

References

1. Kankaanpää, H.T.; Sjövall, O.; Huttunen, M.; Olin, M.; Karlsson, K.; Hyvärinen, K.; Sneitz, L.; Härkönen, J.; Sipiä, V.O.; Meriluoto, J.A.O. Production and sedimentation of peptide toxins nodularin-r and microcystin-lr in the northern baltic sea. *Environ. Pollut.* **2009**, *157*, 1301–1309.
2. Sipia, V.O.; Kankaanpaa, H.T.; Flinkman, J.; Lahti, K.; Meriluoto, J.A. Time-dependent accumulation of cyanobacterial hepatotoxins in flounders (platichthys flesus) and mussels (mytilus edulis) from the northern baltic sea. *Environ. Toxicol.* **2001**, *16*, 330–336.
3. Akter, S.; Vehniäinen, M.; Spoof, L.; Nybom, S.; Meriluoto, J.; Lamminmäki, U. Broad-spectrum noncompetitive immunocomplex immunoassay for cyanobacterial peptide hepatotoxins (microcystins and nodularins). *Anal. chem.* **2016**, *88*, 10080–10087.

© 2017 by the authors. Licensee MDPI, Basel, Switzerland. This article is an open access article distributed under the terms and conditions of the Creative Commons Attribution (CC BY) license (http://creativecommons.org/licenses/by/4.0/).
